# Role of Concomitant Coronary Artery Bypass Grafting in Valve Surgery for Infective Endocarditis

**DOI:** 10.3390/jcm10132867

**Published:** 2021-06-28

**Authors:** Mahmoud Diab, Thomas Lehmann, Carolyn Weber, Georgi Petrov, Maximilian Luehr, Payam Akhyari, Sems-Malte Tugtekin, P. Christian Schulze, Marcus Franz, Martin Misfeld, Michael A. Borger, Klaus Matschke, Thorsten Wahlers, Artur Lichtenberg, Christian Hagl, Torsten Doenst

**Affiliations:** 1 Department of Cardiothoracic Surgery, Jena University Hospital-Friedrich Schiller University of Jena, 07747 Jena, Germany; mahmoud.diab@med.uni-jena.de; 2 Center of Clinical Studies, Jena University Hospital-Friedrich Schiller University of Jena, 07747 Jena, Germany; lehmann.thomas@med.uni-jena.de; 3 Department of Cardiothoracic Surgery, Heart Center of the University of Cologne, 50937 Colonge, Germany; carolyn.weber@uk-koeln.de (C.W.); Maximilian.Luehr@uk-koeln.de (M.L.); thorsten.wahlers@uk-koeln.de (T.W.); 4 Department of Cardiothoracic Surgery, Heinrich-Heine-University Duesseldorf, 40225 Duesseldof, Germany; georgi.petrov@med.uni-jena.de (G.P.); payam.akhyari@med.uni-duesseldorf.de (P.A.); Artur.LIchtenberg@med.uni-duesseldorf.de (A.L.); 5 Department of Cardiac Surgery, Heart Center Dresden, 01307 Dresden, Germany; Sems-Malte.Tugtekin@herzzentrum-dresden.com (S.-M.T.); klaus.matschke@herzzentrum-dresden.com (K.M.); 6 Department of Internal Medicine I, Jena University Hospital-Friedrich Schiller University of Jena, 07747 Jena, Germany; Christian.Schulze@med.uni-jena.de (P.C.S.); Marcus.Franz@med.uni-jena.de (M.F.); 7 Department of Cardiothoracic Surgery, Royal Prince Alfred Hospital, Sydney 2050, Australia; martinmisfeld@yahoo.com; 8 Department of Cardiac Surgery, Heart Center Leipzig, 04289 Leipzig, Germany; Michael.borger@helios-gesundheit.de; 9 Department of Cardiac Surgery, Ludwig Maximilian University Munich, 80539 Munich, Germany; christian.hagl@med.uni-muenchen.de

**Keywords:** valve, endocarditis, concomitant coronary artery bypass grafting, stroke, cardiac surgery

## Abstract

Background: It is current practice to perform concomitant coronary artery bypass grafting (CABG) in patients with infective endocarditis (IE) who have relevant coronary artery disease (CAD). However, CABG may add complexity to the operation. We aimed to investigate the impact of concomitant CABG on perioperative outcomes in patients undergoing surgery for IE. Methods: We retrospectively used data of surgically treated IE patients between 1994 and 2018 in six German cardiac surgery centers. We performed inverse probability weighting (IPW), multivariable adjustment, chi-square analysis, and Kaplan–Meier survival estimates. Results: CAD was reported in 1242/4917 (25%) patients. Among them, 527 received concomitant CABG. After adjustment for basal characteristics between CABG and no-CABG patients using IPW, concomitant CABG was associated with higher postoperative stroke (26% vs. 21%, *p* = 0.003) and a trend towards higher postoperative hemodialysis (29% vs. 25%, *p* = 0.052). Thirty-day mortality was similar in both groups (24% vs. 23%, *p* = 0.370). Multivariate Cox regression analysis after IPW showed that CABG was not associated with better long-term survival (HR: 1.00, 95% CI: 0.82–1.23, *p* = 0.998). Conclusion: In endocarditis patients with CAD, adding CABG to valve surgery may be associated with a higher likelihood of postoperative stroke without adding long-term survival benefits. Therefore, in the absence of critical CAD, concomitant CABG may be omitted without impacting outcome. The results are limited due to a lack of data on the severity of CAD, and therefore there is a need for a randomized trial.

## 1. Introduction

Infective endocarditis (IE) is a serious disease carrying a considerable risk of 1-year mortality [1,2]. Cardiac surgery is required in more than half of patients with IE and is usually indicated when IE is already advanced [3]. The presence of infected tissues makes valve surgery for active IE technically more difficult than for non-IE pathologies. Despite these technical challenges, valve surgery is an independent predictor for better short- and long-term survival in patients with IE [4].

Coronary artery disease (CAD) accompanies the diagnosis of IE in 13–40% of patients and has been identified as an independent predictor of long-term mortality [5]. The current guidelines for patients undergoing aortic valve replacement (AVR) for non-IE pathologies recommend performing CABG to all significant coronary stenoses [6]. This rationale is also applied in IE. However, in patients with IE, a risk and benefit evaluation may be different and may require different strategies to achieve optimal results.

The main cause of death in IE is septic multi-organ failure, which is more likely to occur if clamp and bypass times are long [3,7,8,9]. Adding CABG to valve surgery prolongs the duration of the cardiopulmonary bypass (CPB) and aortic cross-clamping [10,11,12,13]. In addition, the prognostic impact of adding CABG to valve surgery has even been questioned for non-IE patients [14,15]. Importantly, there are no data on concomitant CABG in IE, not even from large registries [1].

We therefore aimed to address the role of concomitant CABG in valve surgery for IE. We retrospectively analyzed data from the Clinical Multicenter Project for Analysis of Infective Endocarditis in Germany (CAMPAIGN), comprising 4917 surgically treated IE patients from six cardiac surgery centers.

## 2. Methods

### 2.1. Patient Population

This retrospective study used data from the CAMPAIGN register, which included all patients who underwent valve surgery for IE between 1994 and 2018 in 6 German cardiac surgery centers.

### 2.2. Data Collection

Data collection was performed under approval by the Institutional Review Board of each participating center. Individual informed consent was waived because of the retrospective nature of the pseudo-anonymized collected data. Long-term follow-up was obtained by review of hospital medical records and interview of patients or their physicians.

### 2.3. Outcome Definitions 

The primary endpoints of this study were 30-day mortality and early postoperative stroke, occurring during hospital stay. Secondary endpoints were rate of re-exploration, postoperative hemodialysis, duration of ventilation, and the duration of intensive care unit and hospital stay, as well as 10-year survival.

### 2.4. Statistical Analysis

Categorical variables are presented as count (valid percentage, excluding missing values). Continuous variables are expressed as mean ± standard deviation (SD) or median (interquartile range) according to their distribution. Student’s *t*-test was used to compare normally distributed continuous variables and the Mann–Whitney-U-test was used for variables not normally distributed. The chi-square and Fisher exact tests were used to compare categorical variables. We performed an inverse probability weighting (IPW) to adjust for differences in basal characteristics between patients with CAD who received CABG and those with CAD who did not receive CABG. The covariate balance after IPW adjustment was assessed by calculating the absolute standardized mean differences (SMD). SMD < 0.1 after IPW adjustment was taken to suggest successful balance achievement between the two groups [16].

The Wald test was used to test for differences in hazards of long-term mortality between two groups. In addition, the hazard ratio (HR) with 95% confidence interval was reported.

To evaluate the influence of concomitant CABG on survival in patients with CAD, a multivariable Cox regression analysis with stepwise regression (backward elimination) was applied. Variables with a *p* value ≤ 0.1 remained in the model. The level of significance was set for all analyses at 5%. All statistical analyses were performed using SPSS Statistics 22 software (IBM Corp., Armonk, NY, USA) as well as SAS 9.4 (SAS Institute, Cary, NC, USA).

## 3. Results

Among 4917 included patients, 615 underwent concomitant CABG and valve surgery and 4302 underwent isolated valve surgery. Table 1 shows the baseline characteristics of all patients divided into those who received concomitant CABG and those who did not receive CABG. Patients who received concomitant CABG were significantly older (68.5 ± 9.5 vs. 61.3 ± 15.0, *p* < 0.001), had higher EuroSCORE (21.12 ± 20.84 vs. 15.43 ± 16.59, *p* = 0.002), more comorbidities, and more frequent pre-operative stroke (29% vs. 21%, *p* < 0.001) compared to patients with isolated valve surgery.

**Figure 1:**
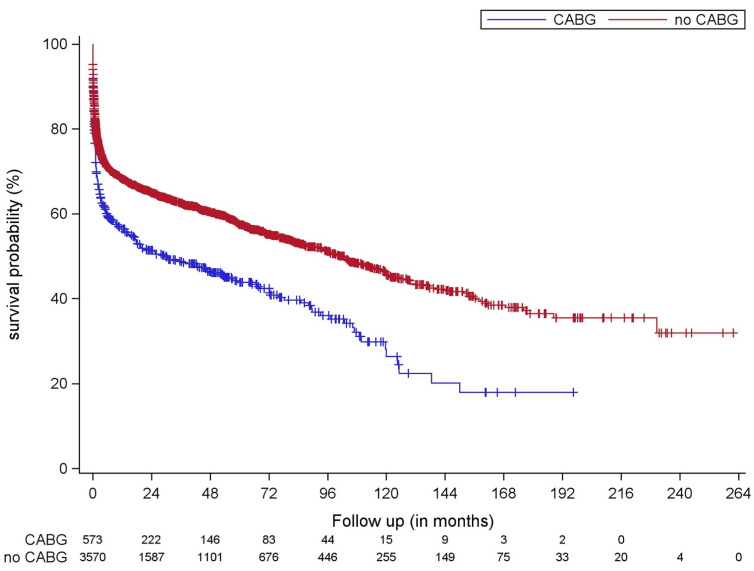
Kaplan–Meier estimates for survival of patients with concomitant CABG (blue line) compared to those without CABG (red line). Concomitant CABG was associated with worse survival (HR: 1.51, 95% CI: 1.33–1.71, log rank < 0.001). CABG: coronary artery bypass grafting; CI confidence interval; HR: Adjusted hazard ratio.

Figure 1 shows Kaplan–Meier survival estimates of patients with concomitant CABG compared to those without CABG in the whole population. The median follow-up time was 14.0 (interquartile range (IQR): 0–55) months. During follow-up, 55% of patients in the group with concomitant CABG vs. 40% of patients in the group without CABG died. Median survival time was 29.0 months (95% confidence interval (CI): 11.7–46.3) in the CABG group and 103.0 months (95% CI: 91.5–114.5) in the group without CABG. Concomitant CABG was associated with worse survival (HR: 1.51, 95% CI: 1.33–1.71, log rank < 0.001).

CAD was reported in 1242 (25%) patients. Among them, 527 patients received CABG and 715 patients did not. Baseline characteristics and operative data for patients with CAD divided into CABG and no-CABG groups are shown in Table 2. Patients who received CABG had more hypertension (73 vs. 68%, *p* = 0.001), hyperlipidemia (38% vs. 32%, *p* = 0.044), PAD (21%, vs. 14%, *p* = 0.002), and more IE of the mitral valve (50% vs. 43%, *p* = 0.01), while patients without CABG had prosthetic IE (46% vs. 17%, *p* < 0.001) and previous cardiac surgery more frequently (54% vs. 21%, *p* < 0.001). The incidence of postoperative stroke was higher in patients with concomitant CABG (27% vs. 20%. *p* = 0.003). Thirty-day mortality was similar in both groups. 

Inverse probability weighting (IPW) was used to compensate for the differences in basal characteristics between patients with CAD who received or did not receive concomitant CABG. Supplementary Figure S1 shows the distribution of the inverse probability score between the two groups, which shows their equality. Table 3 shows the basal characteristics of patients with CAD after IPW. The absolute standardized difference after IPW was greater than 0.10 for only one covariate, which indicates a good matching result. 

Table 4 shows operative procedures and outcomes of the two groups after IPW. Patients who received concomitant CABG had significantly higher incidence of postoperative stroke (26 % vs. 21%, *p* = 0.003). The need for postoperative hemodialysis was also higher in patients who received CABG (29% vs. 25%, *p* = 0.052); however, the difference was not statistically significant.

**Figure 2:**
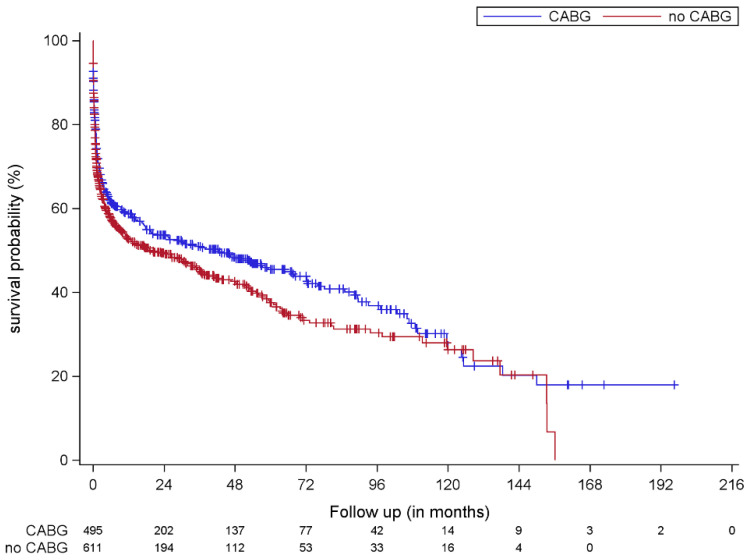
Kaplan–Meier survival estimates of all patients with coronary artery disease comparing those who received concomitant CABG (blue line) to those who did not receive concomitant CABG (red line). CABG: coronary artery bypass grafting.

Figure 2 shows the Kaplan–Meier survival estimates, without IPW, for patients with CAD. Concomitant CABG was associated with better survival compared to no CABG (log rank *p* = 0.047). After IPW, the multivariate Cox regression analysis showed that concomitant CABG was not an independent predictor of better survival (HR: 1.00, 95% CI: 0.82–1.23, *p* = 0.998). Table 5 shows risk factors for mortality among patients with CAD by multivariate Cox regression analysis during the follow-up period. Higher body mass index (BMI), prosthetic valve endocarditis and staphylococcal IE were independent predictors for mortality during the follow-up period.

## 4. Discussion

In this multi-center retrospective analysis, we demonstrate that in the overall study population, concomitant CABG in valve surgery for IE was associated with worse short- and long-term outcomes. Among patients with CAD and after adjusting for the differences in basal characteristics, concomitant CABG was associated with higher incidence of postoperative stroke and a trend to more postoperative hemodialysis without adding a survival benefit. However, a key limitation in our study is the inability to provide information about the severity of CAD and the indications of CABG based on imaging findings. Yet, one still may conclude that if CAD is not considered critical, omitting bypass grafting do not seem to result in inferior outcomes. Because of this uncertainty, the results of this analysis call for a randomized trial.

While cardiac surgery for IE can improve outcomes of patients, it is still associated with high perioperative morbidities and mortality [3]. One of the main explanations for this fact lies in the induction of a systemic inflammatory response to the CPB, which is significantly aggravated in the presence of IE and may progress to multiple organ dysfunction syndrome (MODS) and death in a significant fraction of patients [8,17,18]. We previously showed that septic shock resulting in MODS was the cause of death in 88% of patients who died after cardiac surgery for IE [19]. In addition, valve surgery in the setting of IE is often more complicated than in non-IE pathologies due to multiple valves being affected by IE and the fragility of infected tissues. Thus, adding CABG to such complex surgeries prolongs the duration of CPB and aortic cross clamp times which are known independent predictors of perioperative mortality in patients undergoing cardiac surgery for non-IE [10], as well as for IE [11,12]. In our study, concomitant CABG was indeed associated with longer CPB and cross-clamp durations, and higher in-hospital mortality in the overall patient population. In patients with CAD and after IPW, concomitant CABG was still associated, in addition to the prolonged CPB and cross-clamp durations, with higher rates of perioperative stroke. This higher incidence of perioperative stroke may be due to a greater atherosclerotic disease burden and possibly more manipulation of the aorta in patients who received CABG, or may be due to the prolongation of CPB. One of the major concerns in cardiac surgery for IE is postoperative neurological exacerbation due to hypotension and total heparinization during CPB [20].

The current guidelines for patients undergoing aortic valve replacement (AVR) for non-IE pathologies recommend performing CABG in all significant coronary stenoses with evidence level C [6]. These recommendations are based on data from four retrospective observational studies on patients operated between 1965 and 1986 [21,22,23,24]. In one of these studies, there was no control group without concomitant CABG [21]. In the other three studies, survival was significantly lower in patients with CAD (whether with or without concomitant CABG). Even the presumed long-term benefit of concomitant CABG in patients undergoing AVR for non-IE pathologies has recently been questioned [14,25]. Malberg et al. showed that major adverse cardiovascular events (MACE) in hospital survivors after surgical AVR (*n*=6,870) were similar with or without CABG. They also found that myocardial infarction was more common in patients who received CABG [14]. Our results are consistent with those of Malberg et al., here applied to patients with IE. In the overall study population, concomitant CABG was associated with higher perioperative morbidity, and mortality and worse long-term survival. However, this difference may be due to the underlying CAD and not due to conducting CABG itself. Thus, we investigated IE patients with CAD. After risk adjustment, we found that concomitant CABG was also associated with significantly higher incidences of postoperative stroke and a trend towards higher need for postoperative hemodialysis, without adding a long-term survival benefit. However, since we do not have information on the nature of the CAD, we possibly missed significant difference between the patients in the two groups.

Treatment effects of CABG and PCI as invasive therapies of CAD are currently hotly debated, as a life prolonging effect has recently been questioned by the “Initial Invasive or Conservative Strategy for Stable Coronary Disease” (ISCHEMIA trial) [26]. While the ISCHEMIA trial may only represent a small and very selective patient population [27], a large meta-analysis of all available evidence just demonstrated cardiac survival effects for revascularization (PCI and CABG combined) [28]. We reviewed the available evidence and linked survival impacts associated with CABG or PCI to mechanisms that prevent the occurrence of new myocardial infarctions or reperfuse ischemic myocardium [29,30]. Translating this perspective to IE patients means that CABG may be omitted if CAD is not critical and does not raise concern with getting off the pump and experiencing ischemia in the perioperative period. Most patients with IE do not present with classic symptoms of CAD and therefore do not require symptomatic treatment. Thus, the concern regarding the new onset acute perioperative ischemia may be most relevant. Many CAD lesions only become flow-relevant under conditions of stress. Most imaging methods are geared at detecting stress-induced ischemia. However, theses imaging modalities are often not available in patients presenting with IE. Thus, if the surgeon is concerned that leaving CAD untreated may increase the risk of perioperative ischemia, CABG appears to be in order. However, if this is not the case, our data suggest that CABG may be omitted without adding harm. It is likely that this behavior has been the basis for decision-making in the current data set, which is exactly the reason why a randomized trial is required. It needs to distinguish the risks of surgery in the context of IE with the long-term benefits of CABG, which are in this context further affected by increased risks of IE recurrence and possible limitations of long-term survival from other IE side effects such as stroke, bleeding, or renal dysfunction.

## 5. Limitations of the Study

The key limitation of this study is the lack of data on the severity of stenosis of the coronary arteries; therefore, these data were not included in the IPW. Our cohort may be influenced by referral bias because most participating institutions are tertiary centers. There might be potential bias related to different treatment strategies between participating centers. As in most retrospective multicenter studies, there might be heterogeneity in defining variables and the variables collected may not allow the correct assessment of current risk scores (e.g., EuroSCORE II). However, the results are striking and an important as hypothesis-generating information that challenges a current paradigm which is solely experience-based. It should, therefore, result in the design of a multi-center randomized trial.

## 6. Conclusions

In endocarditis patients with CAD, adding CABG to valve surgery may be associated with a higher rate of postoperative stroke without adding long-term survival benefits. Therefore, in the absence of critical CAD, concomitant CABG may be omitted without impacting outcome. The results are limited due to a lack of data on the severity of CAD and, therefore, there is a need for a randomized trial.

## Figures and Tables

**Table 1:** Baseline characteristics of all patients divided into patients who received CABG and those who did not receive CABG.

Variables	CABG (*n* = 615)	No-CABG (*n* = 4302)	*p*
Age (year)	68.5 ± 9.5	61.3 ± 15.0	<0.001
Male sex	453 (74)	3103 (72)	0.441
BMI	27.3 ± 10.5	26.5 ± 5.8	0.012
EuroSCORE	21.1 ± 20.8	15.4 ± 16.6	<0.001
LVEF (%)			0.078
≥50	429 (70)	3065 (74)	
30–50	158 (26)	939 (23)	
<30	28 (4.6)	146 (3.5)	
NYHA ≥ III	356 (58)	1954 (57)	0.627
Diabetes	227(37)	1285 (26)	<0.001
Hypertension	445 (72)	2047 (48)	<0.001
COPD	87 (14)	428 (10)	0.002
Hyperlipidemia	215 (38)	739 (18)	<0.001
PAD	117 (19.0)	260 (6.0)	<0.001
CAD	527 (86)	715 (17)	<0.001
1-vessel CAD	182 (30)	296 (7)	
2-vessel CAD	158 (26)	182 (4)	
3-vessel CAD	141 (23)	180 (4)	
unclassified	45 (7)	57 (1)	
Pre-operative Stroke	175 (29)	913 (21)	<0.001
Renal insufficiency	284 (46)	1600 (37)	<0.001
Prosthetic IE	136 (22)	118 (28)	0.005
Previous cardiac surgery	157 (26)	1340 (31)	0.005
IE localization			
Aortic	392 (64)	2759 (64)	0.857
Mitral	299 (49)	1879 (44)	0.021
Tricuspid	23 (4)	256 (6)	0.025
Microbiological findings			<0.001
Staphylococcus	209 (44)	1051 (41)	
Streptococcus	88 (18)	704 (27)	
Enterococcus	101 (21)	440 (17)	
Other	83 (17)	371 (15)	

Values are mean ± standard deviation (SD) or *n* (%); BMI: body mass index; CABG: coronary artery bypass grafting; COPD: chronic obstructive pulmonary disease; EuroSCORE: European System for Cardiac Operative Risk Evaluation; IE: infective endocarditis; LVEF: left ventricular ejection fraction; NYH: New York Heart Association functional class; PAD: peripheral arterial disease; CAD: coronary artery disease.

**Table 2:** Baseline characteristics as well as perioperative data and outcome for patients with CAD divided into patients who received (*n* = 527) and those who did not receive concomitant CABG (*n* = 715).

Variables	CABG	No-CABG	*p*
	(*n* = 527)	(*n* = 715)	
Age (yr)	68.8 ± 9.0	69.3 ± 9.5	0.342
Male sex	396 (75%)	599 (78%)	0.221
BMI	27.3 ± 11.1	26.9 ± 4.8	0.371
EuroSCORE	21.4 ± 20.6	22.0 ± 21.5	0.519
LVEF (%)			0.101
≥50	364 (70%)	448 (64%)	
30–50	139 (26%)	203 (29%)	
<30	24 (5%)	48 (7%)	
NYHA ≥ III	316 (60%)	366 (63%)	0.239
Diabetes	196 (37%)	294 (41%)	0.177
Hypertension	384 (73%)	457 (68%)	0.001
COPD	78 (15%)	98 (14%)	0.621
HYPERLIPIDEMIA	185 (38%)	212 (32%)	0.044
PAD	111 (21%)	102 (14%)	0.002
Pre-operative Stroke	145 (28%)	156 (22%)	0.023
Renal insufficiency	249 (47%)	360 (50%)	0.301
Prosthetic IE	89 (17%)	330 (46%)	<0.001
Previous cardiac surgery	108 (21%)	386 (54%)	<0.001
IE localization			
Aortic	319 (61%)	464 (65%)	0.122
Mitral	265 (50%)	306 (43%)	0.010
Tricuspid	19 (4%)	41 (6%)	0.107
Microbiological findings			0.091
Staphylococcus	176 (43%)	192 (46%)	
Streptococcus	74 (18%)	85 (20%)	
Enterococcus	90 (22%)	94 (23%)	
Other	70 (17%)	46 (11%)	
Aortic valve surgery	334 (63%)	481 (67%)	0.165
Mitral valve surgery	279 (53%)	339 (48%)	0.058
Tricuspid Valve surgery	35 (7%)	73 (10%)	0.032
Number of valves			0.041
Single-valve surgery	396 (76%)	533 (76%)	
Double-valve surgery	120 (23%)	153 (22%)	
Triple-valve surgery	4 (1%)	19 (3%)	
Ascending or aortic root	81 (15%)	128 (18%)	0.250
Cross-clamp time (min)	100.29 ± 43.94	87.83 ± 11.19	<0.001
CPB time (min)	153.28 ± 11.12	140.38 ± 72.17	0.003
Length of ventilation (h)	140.29 ± 233.28	146.74 ± 256.28	0.670
ICU stay (d)	8.23 ± 12.39	8.06 ± 11.19	0.800
Hospital stay	16.82 ± 14.21	18.02 ± 17.39	0.227
Postop. Hemodialysis	145 (28%)	164 (24%)	0.125
Postoperative stroke	123 (27%)	124 (20%)	0.003
Re-exploration	87 (17%)	111 (16%)	0.348
30-d mortality	110 (21%)	168 (24%)	0.163

Values are mean ± standard deviation (SD) or *n* (%); BMI: body mass index; CABG: coronary artery bypass grafting; COPD: chronic obstructive pulmonary disease; CPB: cardiopulmonary bypass; EuroSCORE: European System for Cardiac Operative Risk Evaluation; ICU: intensive care unit; IE: infective endocarditis; LVEF: left ventricular ejection fraction; NYH: New York Heart Association functional class; PAD: peripheral arterial disease.

**Table 3:** Baseline characteristics of patients with CAD after inverse probability weighting.

Variables	CABG	No-CABG	*p*	SMD
	(*n* = 527)	(*n* = 715)		
Age (yr)	69.0	69.1	0.822	−0.011
Male sex	76%	77%	0.882	0.024
BMI	26.8	26.9	0.636	−0.021
EuroSCORE	24.74	23.00	0.043	0.092
LVEF (%)			0.252	
≥50	68%	66%		0.002
30–50	27%	28%		−0.022
<30	5%	6%		−0.044
NYHA ≥ III	60%	63%	0.085	−0.004
Diabetes	35%	40%	0.007	−0.008
Hypertension	68%	67%	0.421	0.001
COPD	14%	14%	0.903	0.000
HYPERLIPIDEMIA	38%	36%	0.572	0.003
PAD	17%	17%	1.00	0.000
Pre-operative Stroke	26%	25%	0.810	0.002
Renal insufficiency	44%	49%	0.016	−0.007
Prosthetic IE	32%	33%	0.653	−0.002
Previous cardiac surgery	38%	39%	0.730	−0.002
IE localization				
Aortic	66%	61%	0.03	0.006
Mitral	46%	46%	0.866	0.000
Tricuspid	3%	7%	<0.001	−0.018
Microbiological findings			<0.001	
Staphylococcus	39%	45%		−0.009
Streptococcus	19%	23%		−0.009
Enterococcus	23%	23%		0.000
Other	19	10	<0.001	0.258

Values are mean or %. BMI: body mass index; CABG: coronary artery bypass grafting; COPD: chronic obstructive pulmonary disease; SMD: absolute standardized mean difference; EuroSCORE: European System for Cardiac Operative Risk Evaluation; IE: infective endocarditis; LVEF: left ventricular ejection fraction; NYHA: New York Heart Association functional class; PAD: peripheral arterial disease.

**Table 4:** Operative procedures in patients with coronary artery disease after adjustment using inverse probability weighting.

Variables	CABG	No-CABG	*p*
	(*n* = 527)	(*n* = 715)	
Mitral valve surgery	51%	49%	0.557
Aortic valve surgery	69%	64%	0.011
Tricuspid Valve surgery	6%	9%	0.008
Number of valves			<0.001
Single-valve surgery	74%	77%	
Double-valve surgery	26%	21%	
Triple-valve surgery	1%	2%	
Ascending or aortic root	22%	15%	<0.001
Cross-clamp time (min)	106.1	85.3	<0.001
CPB time (min)	165.9	133.4	<0.001
Length of ventilation (h)	147.2	146.2	0.931
ICU stay (d)	8.3	8.0	0.458
Hospital stay	16.5	17.3	0.266
Postop. Hemodialysis	29%	25%	0.052
Postoperative stroke	26%	21%	0.003
Re-exploration	17%	15%	0.228
30-d mortality	24%	23%	0.370

Values are mean or %. CABG: coronary artery bypass grafting; CPB: cardiopulmonary bypass; ICU: intensive care unit.

**Table 5:** Risk factors for mortality by multivariate Cox regression analysis among patients with CAD during the follow-up period.

Variables	Adjusted HR	95% CI	*p* Value
Age	1.010	0.998–1.022	0.111
BMI	1.026	1.008–1.045	0.005
Hypertension	1.223	0.906–1.651	0.189
Hyperlipidaemia	0.861	0.693–1.070	0.176
Preop. Stroke	1.157	0.915–1.462	0.223
Prosthetic IE	1.339	1.042–1.719	0.023
Staphylococcus	1.257	1.014–1.560	0.037
CABG	1.000	0.815–1.226	0.998

HR: hazard ratio; CI: confidence interval; BMI: body mass index; IE: infective endocarditis; CABG: coronary artery bypass grafting.

## Data Availability

All data are incorporated into the article and its online supplementary material. Additional data will be made available upon request in adherence with transparency conventions in medical research and through requests to the corresponding author.

## Supplementary Materials

The following are available online at https://www.mdpi.com/article/10.3390/jcm10132867/s1, Supplementary Figure S1: The distribution of the inverse probability.
