# Supplementary material for: Role of Concomitant Coronary Artery Bypass Grafting in Valve Surgery for Infective Endocarditis
Source: J Clin Med. 2021 Jun 28;10(13):2867. doi: 10.3390/jcm10132867 (PMC8267636; doi:10.3390/jcm10132867)
Supplement: Supplementary file 1 [file jcm-10-02867-s001.zip › jcm-1231625-supplementary.pdf]

Journal of
Clinical Medicine

SUPPLEMENTARY MATERIALS

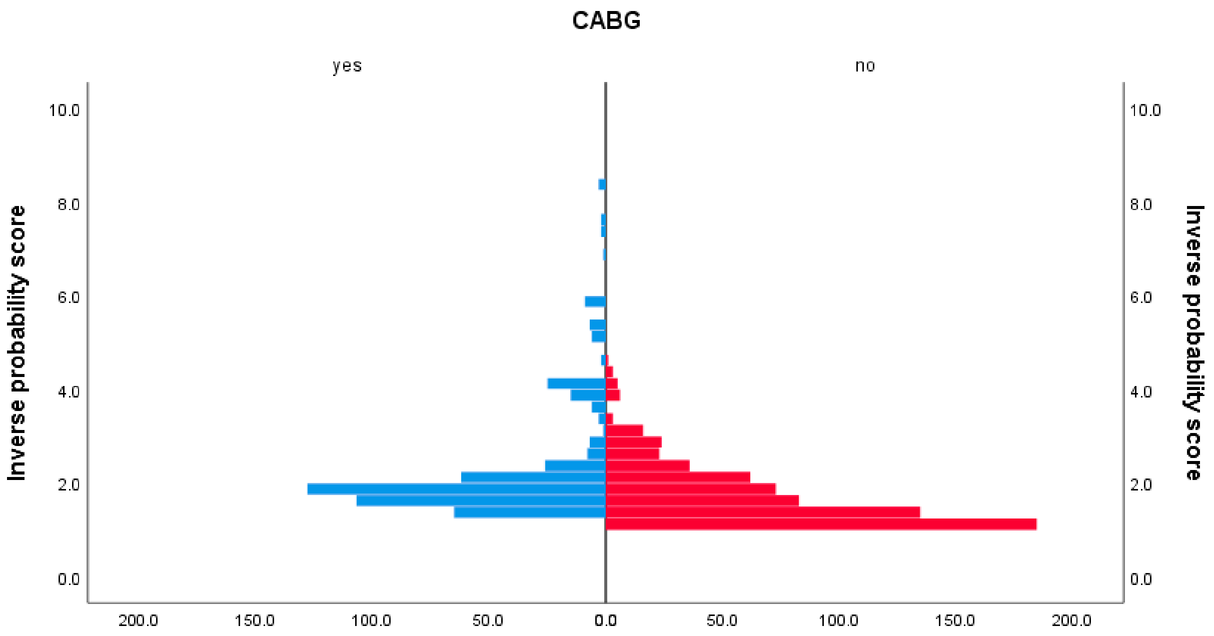

Figure S1. The distribution of the inverse probability score between the two groups. A mirrored histogram showing the distribution of the inverse probability score between patients with coronary artery disease who received concomitant CABG (blue) compared to those with coronary artery disease who did not receive CABG (red). CABG: coronary artery bypass grafting; CAD: coronary artery disease.
